# Clearance function of scavenger endothelial cells

**DOI:** 10.1186/1476-5926-2-S1-S22

**Published:** 2004-01-14

**Authors:** Børd Smedsrød

**Affiliations:** 1 Department of Experimental Pathology, Institute of Medical Biology, University of Tromsø, N-9037 Tromsø, Norway

## Historical Considerations and Semantics

In two recent publications, evidence was presented that the endothelial cell of the mammalian liver sinusoid represents a special scavenger type of endothelial cells that is found in all vertebrates [1,2]. These endothelial cells are located in the liver of land-based vertebrates (mammals, birds, reptiles, amphibians), in the heart or kidney of bony fishes, and in the gill of cartilagenous fishes, lamprey and hagfish. In all animal species studied these specialized endothelial cells show an extraordinarily high uptake of soluble waste macromolecules from the circulation. On this basis the term "scavenger endothelial cell" (SEC) was coined to highlight the biological function of these cells. The first hint that these cells carry out an important physiological function in the elimination of waste material from the circulation was obtained in the early 80s when it was shown for the first time that a physiological waste macromolecule, hyaluronan (HA), was avidly and specifically eliminated from the circulation of rats, rabbits and humans in LSEC [3]. The finding that SEC, but not Kupffer cells (KC) were responsible for this RES function came as a surprise because the general understanding at that time was that blood clearance of material that is too large for glomerular filtration (Mw>20.000) would be eliminated mainly by uptake in KC, which were believed to make up the RES of the liver. In the years that followed we and others showed that an array of physiological and foreign soluble macromolecules and colloids were eliminated from the circulation mainly by receptor-mediated pinocytosis almost exclusively in LSEC. Experiments in other vertebrates, in particular the most numerous class, namely bony fishes, have shown that the specificity and mode of uptake is remarkably similar among vertebrates of considerably different phylogenetic age. This finding further justifies the use of the common term SEC to describe these cells in all vertebrates.

To conceive the significance of SEC it is essential to understand the meaning of RES. Clearly, the meaning of RES has changed from when it was first launched by Aschoff [4] in 1924 and until today. A major finding that lead Aschoff to his conclusions was the numerous studies by him and others showing that vital stains are taken up in certain cells of the body. Another very important finding that contributed to the proposal of RES was Metchnikoff's discovery of *phagocytosis *in macrophages about 30 years prior to Aschoff's RES-publication [5].

Metchnikoff's introduction of phagocytosis as a means for cells to take up material from their surroundings offered a way to explain how vital stain was taken up in cells of RES. It should be noted here that Metchnikoff used the term phagocytosis to describe uptake of both large particles and soluble material such as the vital stain carmin. Today we distinguish between *phagocytosis *and *pinocytosis*. Phagocytosis denotes cell eating or uptake of large particles (roughly 1 micrometer and greater), and is mainly associated with the function of the macrophages, also called "professional phagocytes." Pinocytosis, or cell drinking, denotes cellular uptake of soluble material (macromolecules and colloids smaller than 100 nm). Present-day textbooks in cell biology use the concept of receptor-mediated endocytosis as a special form of pinocytosis ("clathrin-mediated or coated pit-mediated pinocytosis"). When we use these terms today, most of us are unaware of the fact that the concept of pinocytosis was introduced as late as in 1931 [6].

Thus, *phagocytosis *was the only term available to biologists to describe cellular uptake during the period from Metchnikoff until 1931. When Aschoff published his paper on RES in 1924 [4] it would take another decade before the concept of pinocytosis was introduced. Even after the introduction of "pinocytosis" and till the present time many scientists use the word phagocytosis to erroneously describe any type of endocytic uptake, including pinocytosis. The type of pinocytosis that prevails in SEC is receptor-mediated endocytosis via clathrin coated pits. In fact, since SEC are probably the most active pinocytic cell in the vertebrate body it deserves the nick-name "professional pinocyte" for the same reason that macrophages are referred to as "professional phagocytes". Reasoning that uptake in RES is exclusively mediated by phagocytosis, just like most of his contemporary colleagues, van Furth [7] in 1972 recommended that RES be removed from the vocabulary and replaced completely by the mononuclear phagocyte, or "macrophage" system (MPS). This recommendation obscured our ability to discover and understand that Aschoff's RES consisted of non-macrophagic, non-phagocytic cells in addition to macrophages. In fact, if the hepatic RES be defined strictly on the basis of distribution of i.v. injected vital stain, as recommended by Aschoff, pinocytosis in SEC would play a far more important role than phagocytosis in KC in the hepatic RES. Using modern methods of identifying the different types of liver sinusoidal cells, combined with the original vital stain method, Kawai, et al. [8], showed that lithium carmine, the most widely used vital stain among the early RES researchers, was taken up mostly by the LSEC of rat. The lesson to be learned from this historical and semantic survey is that RES of vertebrates consists of two major cell types, namely the macrophage, which eliminates particles from the circulation via phagocytosis, and the SEC, that removes soluble macromolecules via a non-phagocytic receptor-mediated endocytosis.

## Scavenger Function of SEC

We know far more about mechanisms of synthesis of macromolecules than the mechanisms of degradation. This is surprising insofar as anabolism and catabolism are equally important in a body at homeostasis. Moreover, focusing more on synthesis than degradation has led many researchers to underestimate the catabolic arm when studying the turnover of proteins. For instance, in liver fibrosis and some other disorders associated with a higher synthesis of connective tissue molecules, it is common to interpret increased blood levels of these to reflect increased synthesis, and therefore increased fibrotic activity. However, the discovery that LSEC represent the major site of elimination of connective tissue molecules from the circulation [9-14], revealed that increased blood concentrations of these substances reflect both the rate of synthesis as well as their elimination in LSEC.

### SEC can bind but not internalize particles via their endocytosis receptors

It has been shown that red blood cells coated with anti-red cell IgG bind avidly to Fc-gamma receptors on both KC and SEC of rat liver [15] (see section on Fc-gamma receptors below). Interestingly, only KC subsequently engulfed these particles, whereas no internalization was observed in LSEC. In a study to compare binding and internalization of free, soluble chains and particles (1 micrometer) of chondroitin sulphate (CS) it was found that LSEC but not KC bound and internalized the soluble form of CS. Although the CS particles bound to both KC and LSEC, only KC were able to internalize them [16]. These two experiments clearly suggest that KC represent the professional phagocyte, and LSEC the professional pinocyte in the mammalian liver RES. This share of work between macrophages and SEC has been found in animal species of all vertebrate classes [2].

### Colloids are cleared mainly by SEC

Colloids represent small particles (in the nm range) that behave both as soluble macromolecules and particles. Due to their small size, colloids are generally recognized by cells as soluble matter, and consequently are pinocytosed. We have observed that colloidal gold particles up to at least 100 nm distribute almost exclusively to LSEC after i.v. injection in rats (unpublished data). At variance from colloidal gold, colloidal carbon, a substance that was frequently used in the past to test the function of RES, is taken up mainly by KC and other macrophages that are exposed to the blood circulation [17]. This was for some time interpreted as evidence that colloidal carbon is phagocytosed by macrophages. A few authors at that time warned that colloidal carbon binds to and activates platelets, with the consequence that the accumulation of carbon in KC was due to phagocytosis of carbon-laden aggregated platelets rather than uptake of free colloidal carbon. The uptake of platelets due to carbon injection is so active that transient thrombocytopenia has been reported by some authors [18,19].

### Endocytosis receptors in SEC and their macromolecular ligands

A surprisingly low number of receptors (defined according to ligand specificity) are responsible for uptake of a large number of different ligands. In mammals only 4 different receptors have been functionally observed in LSEC. Moreover, studies carried out primarily in Atlantic cod and salmon, but also in species from the other vertebrate classes, have revealed that 3 of these receptors are present on SEC of lower vertebrates [2]. Endocytosis receptors on SEC and some of their physiologic ligands are described in the next paragraphs.

The collagen-alpha chain receptor (COLLA-R) was functionally described for the first time in 1984 [9]. This receptor recognizes only free alpha chains, but not native triple-helical collagen, and is thus a receptor that mediates elimination of collagen waste molecules resulting from physiological collagenolysis. Although it is frequently thought that collagen is a stable protein with a rather long half life, this is not the case if one considers the overall turnover of collagen in the body. In fact, several grams of collagen are catabolized per day in a normal human body. Collagen breakdown in the tissues starts with a single clip by vertebrate collagenase, releasing two large pieces of the triple helix. At body temperature these pieces are unstable, resulting in the generation of free alpha chains. A fraction of these collagen alpha chains is degraded locally, some is sequestered in lymph nodes, and the fraction that finally enters the circulation is efficiently endocytosed and degraded by LSEC [20]. The COLLA-R has so far been observed only in SEC. Studies in rat and cod have revealed that the receptor does not distinguish between different types or species origin of collagen [21]. Uptake studies in animal species from all classes of vertebrates indicate the presence of a COLLA-R in all vertebrate SEC [2]. Binding studies in rat LSEC showed that each LSEC expresses 8 ø 10^4 ^receptors with Kd 1.3 ø 10^-9 ^and 3 ø 10^5 ^receptors with Kd 7.1 ø 10^-9 ^[10]. Work is under way in our laboratory to characterize the molecular structure of the vertebrate SEC COLLA-R. The physiological significance of this receptor is obvious: it prevents accumulation of gelatin in the circulation, thus preventing pathological conditions such as autoimmunity and intravascular clotting.

### The mannose-receptor (MANN-R)

Identical to the macrophage MANN-R, the MANN-R of vertebrate SEC eliminates molecules rapidly from the circulation by Ca^++^-dependent recognition of mannose residues at the terminus of glycoconjugates [22]. Although a large number of both physiological and foreign ligands for this receptor have been reported, I focus here on 3 important physiological waste macromolecules that are cleared from the blood by MANN-R mediated uptake in SEC: i) lysosomal enzymes [23], ii) tissue plasminogen activator [24], and iii) carboxyterminal propeptides of type I procollagen [13].

#### i) Lysosomal enzymes

SEC contain very high specific activities of lysosomal enzymes; with some enzymes the activity is even higher in LSEC than in KC [25]. This observation is compatible with the notion that SEC are extremely active in uptake and degradation of circulating waste products, and therefore need high activities of acid hydrolases in their endosomal/lysosomal apparatus. The high specific activities of lysosomal enzymes in SEC is explained by the fact that the cells recruit these enzymes from the circulation [23]. Lysosomal enzymes are constantly being released to the circulation as part of physiological processes. Since these enzymes carry terminal mannose they are readily recognized by the SEC MANN-R and internalized. However, at variance from most other physiological macromolecules, lysosomal enzymes are not readily degraded. They survive the harsh catabolic milieu in the endosomal/lysosomal compartment, and retain this feature even after they have been captured by SEC. Experiments in rats showed that more than 60% of i.v. injected ^125^I-labeled glycosylasparaginase (GAG) was eliminated with a t_1/2 _of about 0.7 min, the remaining 40% being eliminated with a t_1/2 _of approx. 3 min. The enzyme was cleared almost exclusively by the LSEC. Circulatory survival was significantly prolonged when mannose was included in the injected bolus, signifying uptake via the MANN-R. GAG was highly resistant to degradation after uptake, 33% and 20% of the label being detectable in liver after 1 and 2 days, respectively. Studies with pure cultures of rat LSEC confirmed the *in vivo *findings: GAG was endocytosed at a very high rate, about 40% of added ligand being taken up in the cells after only 60 min. of incubation. After 8 h of incubation with ^125^I-GAG no degradation products could be detected. After 24 h only 10% had been degraded. Endocytosis of the enzyme in cultured LSEC was effectively competed for by mannose. Determination of GAG activity in lysates of LSEC showed that 1) the level of endogenous enzyme decreased after 18 h of incubation *in vitro*, and 2) exogenous enzyme was retained intact in LSEC after endocytosis. Consequently, once captured by LSEC, GAG will carry out its catabolic function in the acid intralysosomal environment. Another lysosomal enzyme, alpha-mannosidase, was found to be processed much the same way as GAG in rat LSEC after endocytosis via the MANN-R (unpublished data). Moreover, a recent study in the Atlantic cod showed that circulating lysosomal cod alpha-mannosidase is removed largely by MANN-R mediated endocytosis in SEC, transported to the lysosomes, and reused there for a long time (days) [26]. A recent study showed that the concentration of 8 different lysosomal enzymes in the blood was several-fold higher in MANN-R deficient mice compared to wild-type mice [27].

The high content of lysosomal enzymes in SEC may alternatively be explained by a very high de novo synthesis. Newly synthesized lysosomal enzymes acquire mannose 6-phosphate (M6P) recognition signals that direct them from the trans-Golgi network to the prelysosomal (late endosomal) compartment. Two receptors, a small (46 kD) cation dependent-receptor and a large (300 kD) cation independent M6P receptor, have been shown to be involved in this sorting function in mammalian cells. Both receptors are required for efficient sorting of lysosomal enzymes [28]. This fact, combined with the observation that LSEC carry very low levels of the large M6PR (unpublished data) support the notion that the cells do not make significant amounts of lysosomal enzymes on their own, which favors the hypothesis that they recruit lysosomal enzymes from the circulation.

#### ii) Tissue plasminogen activator (tPA)

Lysis of a fibrin clot (fibrinolysis) is achieved by the active enzyme plasmin obtained by activation of the proenzyme plasminogen by plasminogen activators. The tissue type plasminogen activator (tPA) binds specifically to fibrin, and there increases its activity several-fold and activates the powerful proteolytic enzyme plasmin only at the site of clotting. We found that tPA is effectively endocytosed in rat LSEC via the MANN-R [29]. Subsequent studies *in vivo *and *in vitro *showed that tPA is cleared by a biphasic kinetics: an initial rapid alpha-phase that removes 76% of injected dose with a t_1/2 _= 0.5 min), and a slow beta-phase that removes 24% of the injected dose with a t_1/2 _= 6.3 min). Competition studies provided evidence that the signals for uptake of tPA in LSEC are terminal mannose and galactose, respectively, which are present in the carbohydrate side-chains of tPA [30-33]. Uptake via the LSEC MANN-R was responsible for the rapid alpha-phase, whereas the slower beta-phase was brought about by the galactose, or asialoglycoprotein receptor of hepatocytes. These studies reveal that the SEC MANN-R regulates the fibrinolytic activity by removing blood-borne tPA.

#### iii) Carboxyterminal propeptide of type I procollagen (PICP)

Type I collagen is the most abundant collagen species in many soft tissues and accounts for more than 90 per cent of the organic matrix of mineralized bone. It is synthesized in the form of a larger protein, type I procollagen, which contains relatively long additional sequences at both ends [34]. These sequences, known as the amino- and carboxyterminal propeptides of type I procollagen, are removed by two specific proteinases in the extracellular space. Proper cleavage of the precursor-specific parts of the molecule is a prerequisite for the appropriate assembly of type I collagen molecules into collagen fibers [35]. PICP contains covalently linked high-mannose type oligosaccharide, one branch per polypeptide chain [36,37]. When PICP is cleaved off *en block *from the procollagen molecule, it is found in free form in interstitial fluid, e.g., in healing wounds [38] and also in blood, where its concentration is regarded to reflect type I collagen synthesis in the body, preferably in the skeleton [39]. However, for the correct interpretation of the concentration of PICP in blood it would also be essential to know the routes along which this compound leaves the circulation. We have shown that uptake of PICP is another function of the SEC MANN-R [13]. The same conclusion was recently reached by analyzing the serum protein profile of MANN-R knockout mice [27].

### The hyaluronan/scavenger receptor (HA/S-R)

Studies on the turnover of the connective tissue polysaccharide hyaluronan (HA) show that this substance is eliminated from the circulation almost exclusively by receptor-mediated endocytosis in SEC of rat liver, salmon kidney, and cod heart [2]. A series of experiments performed to determine the exact specificity suggested that rat LSEC express an endocytosis receptor with binding specificity only for HA and the related connective tissue polysaccharide chondroitin sulphate (CS) [40]. The affinity was calculated to be rather high (Kd = 10^-11 ^for HA of Mw 4 ø 10^5^), and the number of receptors expressed per cell was estimated to be about 8000 [40]. Both HA and CS are highly negatively charged polymers, and it was speculated that these molecules be recognized by the so-called scavenger receptor (SR), which is actually a group of different endocytosis receptors with only one feature in common, namely the ability to take up negatively charged macromolecules. To settle this question, receptor-ligand competition experiments were carried out to see if typical SR-ligands were able to inhibit binding and uptake of HA and CS. None of these experiments yielded results to support that HA is taken up in SEC via the SR. However, in 1988 Eskild et al. [41] reported that formaldehyde-treated serum albumin (FSA), a frequently used test ligand for the SR, was able to inhibit the binding and uptake of free chains of CS, but not HA in isolated rat LSEC. Curiously, in cross competition experiments CS was unable to inhibit uptake of FSA. Moreover, neither FSA nor HA competed with each other for uptake. These findings left a hint that the HA and SR were either the same protein or different receptors with overlapping ligand specificities. It was not until McCourt et al. in 1999 published their paper on purification of the rat LSEC HA receptor [42] that the enigma found its solution. These workers used affinity purification, with either HA or aminoterminal propeptide of type I procollagen (PINP), a protein that is cleared exclusively by the SR of rat LSEC (10, and below), and showed that a LSEC surface protein of identical Mw (~200 and ~350 kD) was purified irrespectively of whether HA or PINP was used. Using antibodies generated against this protein, it was possible to inhibit endocytosis of both HA, and various SR-ligands [42]. The inability of a number of SRs to inhibit endocytosis of HA was explained by binding affinity, which increases with the chain length of the polysaccharide. The reason why Eskild et al. observed that FSA inhibited CS but not HA [41] was most likely that the CS chains used were short (only about 10ø20 kd, which would bind with an estimated Kd of 10^-6 ^ø 10^-7^), whereas the HA chains were in the order of 10^6 ^d (with a Kd of about 10^-11^). Thus, the binding of these short CS chains to the receptor was relatively weak, whereas the binding affinity of the much longer HA-chains was 4ø5 orders of magnitudes higher. Since the competing ligand, FSA, bound to the receptor with a Kd of about 10^-9^, it would compete strongly with CS, but not with HA for binding to LSEC.

The SR function was discovered on macrophages, and has been attributed to the accumulation of cholesterol-laden modified LDL and thus initiate a series of inflammatory events in the intima of the vessel wall, leading to atherosclerosis [43]. Today several SRs are known, many of which have been characterized and cloned. The first SR to be cloned was the macrophage SR, or SR-A [44]. This receptor has been found on SEC, but reports showing that transgenic mice lacking this receptor still remove SR-ligands from the circulation as avidly as do wild-type mice, suggest that these macromolecules are taken up from the circulation by a different SR [45,46]. In fact, *in vitro *cultures of SEC from SR-A knock out mice showed the same uptake rate and capacity of endocytosis of several SR-ligands [46]. These results, along with the recent characterization by McCourt et al. of the HA/S-R [[42], and McCourt's article in this series of online proceedings], show that LSEC, employing this unique receptor, represents the most important cellular site of uptake of blood-borne SR-ligands. The mouse and human forms of HA/S-R have now been cloned by Politz et al. [47] (Accession nos. AF364951 and AJ295695) under the name Stabilin-2 (see also McCourt's article in the present proceedings).

It is unfortunate that the name "scavenger receptor" has been taken to name exclusively this group of receptors. When dealing with SEC it is obvious that both the COLLA-R, MANN-R, and Fcgamma-R must be regarded as different types of SRs. The term "scavenger receptor" has no logical connection to the definition presently used ("the receptor that scavenges negatively charged macromolecules"). Semantically and logically it would be better to associate "scavenger receptor" with any clearance receptor that eliminates waste products. Thus, all endocytosis receptors on SEC deserve the name "scavenger receptors".

In the following, I give two examples of categories of waste substances that are eliminated by the SEC HA/S-R: i) N-terminal propeptides of types I and III procollagen (PINP and PIIINP), and ii) Atherogenic molecules: oxidized LDL (oxLDL) and advanced glycation end products (AGEs). Clearance of these substances represents an important physiological mechanism contributing to maintaining homeostasis, and preventing atherosclerosis.

#### i) Aminoterminal propeptides of types I and III collagen (PINP and PIIINP)

Collagen is produced and secreted as procollagen, which consists of large extra domains known as the procollagen propeptides. After secretion procollagen is converted to collagen by specific enzymes, the endopeptidases procollagen N- and C-proteinases [35]. The collagen molecules thus formed assemble into collagen fibrils. It has been generally assumed that these procollagen propeptides, thus released to the extracellular space, would rapidly be eliminated locally. While there may be some local elimination by unspecific endocytosis and degradation, there is now ample evidence that these propeptides are transported with the lymph to the circulation and there rapidly eliminated by efficient uptake in LSEC [20]. *In vivo *and *in vitro *studies in rat have shown that PINP and PIIINP are endocytosed via the SEC HA/S-R [14,42]. Both PINP and PIIINP contain clusters of phosphate groups, and it is likely that these highly negatively charged groups are recognized by the HA/S-R. The body produces several grams of collagen per day. Knowing that one PINP molecule is released per collagen type I molecule that is deposited in the connective tissue, it is evident that the LSEC must have a high capacity to eliminate these collagenous molecules effectively from the circulation. Elimination of the carboxyterminal propeptide of type I procollagen is dealt with under the MANN-R (see above).

#### ii) Atherogenic molecules: oxidized LDL (oxLDL) and advanced glycation end products (AGEs)

Oxidatively modified low density lipoprotein (OxLDL) has been held as a factor that may trigger the development of atherosclerosis. Recently advanced glycation end products (AGEs) have also been suggested as an atherogenic substance [48]. Both OxLDL and AGEs are removed from the circulation by SR-mediated endocytosis in KC and SEC [49,50]. AGE-modified albumin (AGE-BSA) exists in a wide spectrum of molecular sizes, spanning from the smallest (monomers, the size of BSA) and up to highly aggregated polymers. Intravenous administration of AGE-BSA following separation of the molecules into high and low Mw fractions, showed that the highly aggregated AGE-BSA was taken up preferentially in KC, whereas the low Mw specimens were eliminated largely by LSEC [13]. This distribution according to degree of aggregation is compatible with the general share of work between the two cell types, the KC taking up highly aggregated material, and SEC being specialized to take up soluble material. Due to the fact that OxLDL like AGEs represent a rather heterogeneously sized group of molecules, it would be of interest to see if high and low molecular size aggregates of OxLDL distribute in the two types of sinusoidal liver cells in the same way as was observed with high and low molecular size AGE-BSA.

It was recently shown that endocytosis of AGEs leads to depletion of the SR that is responsible for clearance of AGE, namely HA/S-R [51]. In this way AGE downregulates its own removal from the circulation. Since OxLDL is probably also taken up in LSEC via the same HA/S-R, it may be speculated that one of the serious pathogenic effects of AGE may be its tendency to lower its removal, with the consequence that the blood concentration of these and other atherogenic molecules may increase, thus increasing the risk of developing atherosclerosis and other vascular complications.

#### iii) Coagulation products

Serum, but not native blood plasma, contains significant amounts of material that is eliminated by the LSEC SR [52]. This shows that blood coagulation in the presence of platelets generates numerous waste macromolecules that are readily eliminated from the circulation by receptor-mediated endocytosis in LSEC. Although these coagulation waste products have not yet been characterized, it has been shown that they represent molecules of a large spectrum of molecular size, and carry a high net negative charge. On this basis it may be speculated that they represent coagulation factors and other blood proteins that have been tagged with negative charges by platelet-mediated phosphorylation. Based on the notion that LSEC represent an important site of elimination of atherogenic molecules, any mechanism that attenuates this activity of LSEC should be viewed as a potentially pro-atherogenic mechanism. Thus, in individuals with a constant activation of platelets ø as in diabetic patients [53] ø it is possible that the increased load of coagulation-waste products compete with the normal scavenging of atherogenic molecules such as oxLDL and AGE.

### Fc-gamma receptor (FcgR)

FcgRs recognize the Fc domain of immunoglobulin G (IgG). These receptors can mediate phagocytosis of large IgG coated particles and pinocytosis of soluble IgG immune complexes. Combined Northern analysis and RT-PCR-studies have shown that KC and LSEC express the three FcgRs FcgRIII, FcgRIIB1 and FcgRIIB2 [54]. Blood-borne IgG-immune complexes are taken up almost exclusively in liver, both in KC and LSEC [55,56]. Although IgG immune complexes are removed from the blood predominantly by KC, it has been shown that following KC depletion these immune complexes become strongly associated with LSEC [57]. Cultured KC and LSEC both avidly bind IgG-coated erythrocytes [15]. Since soluble IgG complexes could compete with this binding it was concluded that both cell types bind IgG-coated red cells specifically via FcgRs. In contrast to LSEC, only KC internalized the bound red cells, reflecting the general principle that KC perform phagocytosis of particles >1 micrometer, whereas LSEC internalize only nm size colloids or truly soluble macromolecules.

## Other Functions Related to SEC Scavenger Activity

### Interaction of SEC with virus

It has long been known that SEC of mammalian liver represent an important site of uptake of blood-borne virus, for instance hepatitis virus type 3 in mice, FIV (Feline Immunodeficiency Virus) in cats, and HIV-1 (Human Immunodeficiency Virus type 1) in humans [58-62]. If the virus is sorted intracellularly through a route to avoid degradation in the endosome/lysosome apparatus, it may survive for a long time inside SEC, and/or be transferred to neighbouring hepatocytes and bring about infection and replication there. Recently, experiments with duck hepatitis B virus, which replicates exclusively in hepatocytes, showed that this virus is first endocytosed by LSEC, rescued from the endosomal/lysosomal route and then transferred to the hepatocytes [63].

### Regulation of endocytosis in SEC

A few studies have addressed the regulatory effect of inflammatory mediators on endocytosis in SEC. Martinez et al. [64] found that tumor necrosis factor-alpha and interleukin-1 beta (IL-1) enhance 2ø3-fold endocytosis via the SR and MAN-R, while COLLA-R mediated endocytosis remained unaffected. Lipopolysaccharide was found to increase endocytosis in LSEC indirectly, by stimulating the cells to release autocrine IL-1. Another mediator, nitric oxide decrease endocytosis via the MAN-R in LSEC [65]. Using IL-10, Knolle et al. [66] found a similar effect as that reported with nitric oxide, namely down-regulation of MAN-R mediated endocytosis in SEC.

### Role of SEC in innate and specific immunity

SEC remove not only physiological waste products from the general circulation; material absorbed from the gut, including virus, lipopolysaccharide and other foreign and pathogenic substances, are also efficiently eliminated by these cells [3]. This unique ability to endocytose practically all physiological and foreign blood-borne, soluble waste macromolecules shows that these cells are indeed an important part of the vertebrate innate immune system. It was recently found that LSEC constitutively express all molecules necessary for antigen presentation: CD54, CD80, CD86, MHC class I and II, and CD40, and can function as antigen-presenting cells for CD4^+ ^and CD8^+ ^T cells [67,68]. In this way LSEC function as an organ-resident antigen-presenting cell inducing local specific tolerance in the liver towards soluble blood-borne antigens. Thus LSEC-mediated immune tolerance of liver resides in two unique features of this cell type: i) a scavenger function that removes blood-borne antigens quantitatively by very active endocytosis, and ii) an antigen-presenting activity that induces immune tolerance in CD4^+ ^and CD8^+ ^T cells.

### Role of SEC in liver transplantation and bioartificial liver (BAL)

It is now generally accepted that graft rejection in liver transplantation is often due to malfunctioning LSEC [69]. Donor liver is very sensitive to the reperfusion insults associated with KC activation and production of reactive oxygen radicals, which attack SEC [70,71]. Consistent rise in blood HA is a clear sign that the graft will be rejected [72], supporting the idea that damage to LSEC precedes rejection of grafted liver. Acute liver failure is associated with 40ø60% mortality, and transplantation is frequently the only treatment that can save the patient. A bioartificial liver (BAL) to keep the patient alive until a donor liver is available, has recently been introduced. The most commonly used BAL consists of isolated pig hepatocytes. Liver transplantation and BAL are often studied in pig models. Based on what we presently know about the function of LSEC, it is surprising that all BAL studies so far have dealt with BALs that are made up of hepatocytes only. Moreover, it is a puzzling fact that no publications have so far dealt with the scavenger function of liver in pig. Although one may guess that SEC of pig liver carry out the same scavenger functions as the corresponding cells in rats, it remains to be shown. Two papers presented at this meeting (by Geir Ivar Nedredal and Kjetil Elvevold) show that SEC of pig liver perform the same scavenger function as SEC in rat liver. This is important knowledge for any laboratory that is engaged in BAL, and shows that a BAL that comprises only hepatocytes does not have the ability to remove waste macromolecules from the circulation. In conclusion: A complete, functional BAL depends on the presence of intact SEC. Of note, KC should be avoided from BAL, since those cells are easily activated to produce cytotoxic oxygen radicals that are very harmful to SEC [70,71]

### SEC and energy production

The final degradation products released from both mammalian and fish SEC after endocytosis of HA and CS labeled with ^14^C in the carbon atoms of the pyranose rings or with ^3^H in the acetyl groups have been identified as ^14^C-lactate and ^3^H-acetate [40,73]. These findings, along with the fact that these cells contain few mitochondria, suggest that vertebrate SEC have a largely anaerobic type of metabolism. Recent research in our laboratory shows that SEC produce large amounts of lactate and in this way may supply neighbouring energy-consuming cells with energy rich fuels such as lactate. This opens up for the possibility that SEC represent local power plants that produce high octane fuel.

## Conclusions

In this review on SEC I have focussed on the function that is most typically associated with these cells, namely their scavenger function. It is now clear that SEC of vertebrates carry out many of the uptake functions that had previously been incorrectly ascribed to macrophages. As a consequence the concept and functional significance of RES have been reborn. It is now appreciated that the hepatic RES of mammalia consists of two different cell populations, namely LSEC that eliminate soluble waste colloids and macromolecules, and KC that remove insoluble particles of greater-than colloid size. In all vertebrates the same share of work exists between SEC and macrophages. Studies in phylogenetically distant vertebrate species show that 3 functionally different endocytosis receptors are present in SEC: the COLLA-R, the MAN-R, and the HA/S-R. In addition LSEC of mammals express FcgR. With these few receptors SEC eliminate a large number of blood-borne colloidal and soluble waste macromolecules that enter the liver both from the general circulation and the gut. It is conceivable that SEC play a major role in keeping the blood free of any type of macromolecular waste. While it is obvious that this property of SEC is highly advantageous to maintain a physiological macromolecular composition of blood, the cells represent a major problem in drug targeting whenever an i.v. administered macromolecular drug is intended to reach a tissue different than the liver. That is, if the goal is to target the SEC proper, it will be an easy task to develop a carrier system, since almost any modification of native proteins will make them a ligand for SEC endocytosis receptors. However, if the goal is to target a drug to an extrahepatic target, it takes great skill to design the drug and its vehicle in such a way that it is not recognized by the powerful uptake system in LSEC.

Recent research in our laboratory has shown that vertebrate SEC run a largely anaerobic metabolism. The cells carry few mitochondria, are exposed to blood of low oxygen tension, and generate large amounts of lactate, that may be utilized by neighbouring high energy consuming epithelial cells as fuel for efficient production of ATP.

How important is the scavenger function of SEC for the maintenance of normal physiology in mammals? Can we survive without LSEC? Probably not. The fact is that these cells are utterly important for the liver integrity; the liver would soon stop functioning without them. This can be illustrated by the following three examples: i) Ricin, a highly toxic substance that is removed from the circulation mainly by MAN-R mediated endocytosis in SEC [74], destroys the SEC specifically. About 72 h following i.v. administration of ricin, rats die due to severe liver failure. In comparison, depletion of KC leaves the liver apparently fully intact. ii) A transplanted liver will be rejected if the SEC are damaged [71]. On the other hand, if KC are depleted from the donor liver, it increases the chances that the transplanted liver will not be rejected. iii) The mechanism of acetaminophen (paracetamol) liver intoxication resides in the effect of this drug on LSEC [75]: Experiments in mice revealed that massive injury to SEC and thereby disrupted the microcirculation of liver preceded development of liver failure [76].

Research over the past 20ø30 years on the endothelial cell that comprises the wall of the mammalian liver sinusoid has revealed that these cells represent a far more important part of the liver than just forming the wall of the sinusoids. New knowledge about the functions of these cells leave no doubt that they represent a functional part of liver that we cannot do without. The cells remove an array of waste macromolecules, including virus from the blood. Recent evidence suggests that this scavenger function is uniquely coupled to production of lactate which supplies the hepatocytes with high octane fuel. It is also evident that the cells represent a dendritic-like type of cells that present antigen to invoke specific immune tolerance. Recent research shows that mammalian LSEC represent a member of the vertebrate SEC system, which together with macrophages comprise the backbone of the vertebrate RES. It is time to realize that liver biology cannot be understood without the knowledge of the physiological function of LSEC.
